# Barriers and facilitators to pre-exposure prophylaxis uptake among male sex workers in Mexico: an application of the *RE-AIM* framework

**DOI:** 10.1186/s12889-021-12167-9

**Published:** 2021-11-27

**Authors:** Hemant Kadiamada-Ibarra, Nicola L. Hawley, Sandra G. Sosa-Rubí, Marta Wilson-Barthes, Roxana Rodríguez Franco, Omar Galárraga

**Affiliations:** 1 Brown University School of Public Health, Providence, RI 02912 USA; 2 Department of Chronic Disease Epidemiology, Yale School of Public Health, New Haven, CT 06510 USA; 3 Division of Health Economics, National Institute of Public Health (INSP), 62100 Cuernavaca, CP Mexico; 4 Department of Epidemiology, Brown University School of Public Health, Providence, RI 02912 USA; 5 Center for Demographic, Urban, and Environmental Studies (CEDUA), The College of Mexico, 14110 Mexico City, CP Mexico; 6 Department of Health Services, Policy, and Practice, Brown University School of Public Health, Providence, RI 02912 USA

**Keywords:** Pre-exposure prophylaxis, Men who have sex with men, Male sex workers, Mexico, RE-AIM

## Abstract

**Background:**

The ImPrEP México demonstration project is the first to distribute free HIV pre-exposure prophylaxis (PrEP) to men who have sex with men (MSM) and transgender women living in Mexico. In Mexico City, MSM who are also male sex workers (MSWs) face a disproportionately high risk of HIV infection. PrEP is highly effective for HIV prevention, yet “real-life” implementation among MSWs is a challenge due to the unique adherence barriers faced by this population.

**Methods:**

This study uses the *RE-AIM* implementation science framework to characterize the unique barriers to and facilitators of PrEP uptake among MSWs in Mexico City. We conducted 9 in-depth key informant interviews and 2 focus group discussions with MSWs across 5 clinic and community sites. Qualitative data were analyzed using inductive, open coding approaches from grounded theory. We supplemented findings from the primary qualitative analysis with quantitative indicators derived from ImPrEP program records to describe the current *Reach* of the ImPrEP program among MSWs in Mexico City and the potential for wider PrEP *Adoption* among other high-risk populations in Mexico.

**Results:**

The *Reach* of the ImPrEP program was 10% of known HIV-negative MSWs in Mexico City. Program *Reach* was lowest among MSWs who were street-based sex workers, of lower socioeconomic status, migrants from other states and self-identified as heterosexual. Barriers to program *Reach* included limited PrEP knowledge, HIV-related stigma, and structural barriers; facilitators included in-person program recruitment, patient-centered care, and spread of information through word of mouth among MSWs. Two out of the four eligible institutions had adopted the ImPrEP protocol. Barriers to wider program *Adoption* included HIV- and sexual identity– related stigma, protocol limitations, and lack of a national policy for PrEP distribution; facilitators of *Adoption* included existing healthcare infrastructure, sensitized providers, and community support from non-governmental organizations.

**Conclusions:**

Increasing the ImPrEP program’s *Reach* among MSWs will depend on improving PrEP education and addressing HIV-related stigma and access barriers. Future *Adoption* of the ImPrEP program should build on existing clinical infrastructure and community support. Creation of a national policy for PrEP distribution may improve the *Reach* and *Adoption* of PrEP among highest-risk populations in Mexico.

**Supplementary Information:**

The online version contains supplementary material available at 10.1186/s12889-021-12167-9.

Contributions to the literature

- This study is the first to evaluate barriers to and facilitators of PrEP implementation and adherence among male sex workers in Mexico
- Male sex worker perspectives on public health interventions are lacking in the literature; findings from this study add to the current evidence base surrounding the optimal design of HIV prevention programs for this population
- Application of the *RE-AIM* framework increases the generalizability of study findings to other health interventions targeting hard to reach populations

## Background

Over 9800 new HIV infections and 5800 AIDS diagnoses were reported in Mexico in 2019 [1]. While HIV prevalence is 0.3% among Mexico’s general adult population (15–49 years of age), the burden of the epidemic is concentrated in sub-populations; nearly 1 in every 5 men who have sex with men (MSM) are HIV positive and prevalence among transgender individuals and male sex workers is 17.4 and 18.2%, respectively [2–4]. In Mexico City alone, HIV prevalence estimates among male sex workers (MSWs) are as high as 38% [4].

Individual-level and contextual factors place MSWs at a greatly increased risk of HIV infection. MSWs often receive increased monetary incentives from clients to engage in unprotected sexual behaviors (e.g., condomless anal sex) [5]. MSWs have multiple sexual partners, high rates of drug and alcohol use, and experience psychosocial conditions (e.g., depression), stigma, violence, and housing instability [6]. Furthermore, MSWs often do not know their HIV status due to limited access to care and a paucity of widespread testing [7].

To address the epidemic, Mexico’s Universal Antiretroviral Treatment (ART) Program has been providing free ART for uninsured populations since 2003 [8]. Despite the effectiveness of this longstanding program, HIV incidence is projected to increase by 2030, signaling that treatment alone is not enough to curb the epidemic [9]. Daily oral use of emtricitabine and tenofovir disoproxil fumarate (FTC/TDF) as pre-exposure prophylaxis (PrEP) can reduce the risk of HIV infection by 92% and is recommended by the World Health Organization (WHO) for MSM at risk of HIV infection [10, 11]. Nevertheless, PrEP uptake is primarily used among MSM in high-income countries with limited availability in low- and middle- income countries (LMICs) [12].

In 2018, Mexico launched the UNITAID-funded ImPrEP Project as a preparatory step for widespread integration of PrEP services in the country. ImPrEP México provides free PrEP to a selected sample of MSM and transgender women living in Mexico City, Guadalajara, and Puerto Vallarta, cities which are epicenters of the country’s HIV epidemic [13]. The ImPrEP Project is also operating in Brazil and Peru to address the strategic aspects of PrEP implementation and inform sustainable programs in regions of high HIV prevalence.

In order to be scaled successfully, “real-life” implementation of PrEP programs will need to address the unique adherence barriers faced by MSM and MSWs. In the United States, one of the earliest adopters of PrEP, individual-level factors including lack of awareness, distrust of medical systems and concerns about side effects are barriers to PrEP uptake. At the systems-level, lack of funding and minimal provider education, coupled with HIV-related stigma, transphobia and homophobia reduce PrEP access [14]. These challenges are amplified in LMICs, where competing health system priorities and stigma towards sexual minorities are pervasive. Efforts to bring PrEP to scale in Mexico must address the unique PrEP adherence barriers faced by those at highest risk of HIV acquisition.

This study uses an implementation science framework to identify the unique barriers to and facilitators of a PrEP adherence program for male sex workers in Mexico City. To the best of our knowledge, this is the first study to evaluate the implementation of a PrEP intervention among the population of MSWs in Mexico.

## Methods

Our study uses the *Reach*, *Effectiveness*, *Adoption*, *Implementation*, and *Maintenance (RE-AIM)* implementation science framework [15] to characterize barriers and facilitators to Pre-Exposure Prophylaxis use among MSWs in Mexico. The RE-AIM framework was first conceived in 1999 to evaluate and improve the external validity and sustainability of public health interventions. Applications of the framework have historically emphasized descriptive or quantitative data related to physical activity, obesity, and disease management programs, with increasing focus on qualitative RE-AIM assessments [15]. There is also growing support for more pragmatic applications of the framework that address only select RE-AIM components – rather than all five dimensions – that are most relevant for a specific research question or stakeholder group [16, 17].

In this study, we use the RE-AIM framework to evaluate the *Reach* of the ImPrEP México demonstration project among MSWs in Mexico City and the potential for wider *Adoption* of PrEP among other high-risk populations in Mexico. *Reach* and *Adoption* were selected for this analysis because they are arguably the most important RE-AIM components for informing demand for and longer-term adoption of a novel demonstration project. *Reach* is defined as the absolute number, proportion, and representativeness of individuals who are willing to participate in a given initiative, intervention, or program. *Adoption* is defined as the absolute number, proportion, and representativeness of settings who are willing to initiate a program [18].

We use a primarily qualitative approach to data collection and augment qualitative findings with quantitative indicators of *Reach* and *Adoption* ascertained from program records.

### Qualitative data collection

Qualitative data were obtained from semi-structured interviews with key informants who were implementing the ImPrEP México protocol in Mexico City and via focus groups with MSWs in Mexico City.

#### Key informant interviews

Key informant interviews were conducted in person from June to August 2019 by research personnel from Brown University. Key informants were identified through established networks between researchers at Brown University, Mexico’s National Institute of Public Health (INSP), and Clínica Especializada Condesa, Mexico’s largest specialty HIV prevention and care government clinic. All key informants were chosen based on their involvement in the organization or the execution of the ImPrEP México project in Mexico City. A combination of purposive and snowball sampling was used to select 9 key informants for semi-structured interviews: 5 key informants were selected from Clínica Condesa, 2 from Fundación México Vivo and 2 from Inspira Cambio. Fundación México Vivo and Inspira Cambio are non-governmental organizations (NGO) working to address the burden of HIV/AIDS in the country. All key informants from Clínica Condesa were invited in person. Key informants at Fundación México Vivo and Inspira Cambio were invited via email following recommendations by Clínica Condesa key informants.

Interviews were conducted in private office spaces at the work site of each informant. At Clínica Condesa, all interviews were conducted individually. At the other sites, participants were interviewed together for efficiency purposes as requested by the key informants. Prior to each interview, each key informant provided written informed consent and received an information packet explaining the *RE-AIM* framework and study objectives. All interviews were conducted in person by research personnel trained in qualitative interview techniques. All interviews were conducted in Spanish using a semi-structured interview guide developed explicitly for this study, lasted approximately 45–60 min, and were audio recorded. (English language version of the interview guide is provided in Supplementary Material 1).

#### Focus group discussions

Two focus groups were conducted with a total of 8 male sex workers who were not enrolled in the ImPrEP México. (Table 1) Following Liamputtong et al., focus group methodology is “suitable for examining sensitive issues and for research involving vulnerable and marginalized populations because people may feel more relaxed about talking about these issues when they see that others have similar experiences or views.” [19]

**Table 1:** Descriptive characteristics of male sex workers who participated in focus group discussions

	Focus Group Participants *N* = 8
Age in years, mean (SD)	30.3 (6.8)
Current place of residence, *n*(%)	
Mexico City	7 (87.5)
Outside Mexico City in Mexico State	1 (14.3)
Housing status, *n*(%)	
Renting a house or apartment	3 (37.5)
Owns own residence	2 (25.0)
Living with family	1 (14.3)
Living on the street	1 (14.3)
Living in a hotel	1 (14.3)
Highest level of schooling completed, *n*(%)	
Secondary	2 (25.0)
High school or technical career training	4 (50.0)
College	1 (14.3)
Missing	1 (14.3)

*SD* standard deviation

Focus groups were conducted in January 2020 at two community sites in Mexico City, the Community Center for Attention to Sexual Diversity and the Condomóvil offices [20, 21]. The focus groups aimed to: (1) explore the experiences of MSWs in regard to HIV risk behaviors, (2) promote awareness of PrEP and other HIV prevention services, and (3) guide the development of PrEP education materials targeted to this population. MSWs were recruited using convenience sampling by staff from Condomóvil. Recruitment was conducted in the Zona Rosa and Alameda neighborhoods of Mexico City where sex work is common. Condomóvil staff explained the study objectives to participants and emphasized that participation was voluntary and all responses would be kept anonymous. MSWs who agreed to participate provided their contact information and were offered transportation to and from the focus group.

Both focus groups were conducted by male and female clinical staff from Clínica Condesa and Roxana Rodríguez-Franco, MPH (female, co-author) from Mexico’s National Institute of Public Health. All group facilitators had prior experience conducting focus groups with populations at increased risk of HIV infection, mainly MSWs and transgender women. At the start of each focus group discussion, staff repeated the study objectives and rules of participation. Participants provided written informed consent and completed a sociodemographic questionnaire. Participants could use pseudonyms to protect their anonymity. Discussions were conducted in Spanish using prompts specific to the *Reach* of PrEP services in Mexico City. (Supplementary Material 1) Discussions lasted approximately 2 h with a 20-min break and were audio-recorded.

Study protocols for focus group discussions were reviewed and approved by the National Institute of Public Health (INSP) Ethics Committee in Cuernavaca, Mexico (CI/2019/147). All key informants and focus group participants provided written informed consent for study participation. Qualitative data collection followed the Consolidated Criteria for Reporting Qualitative (COREQ) Research checklist.

### Data collection for quantitative indicators

To supplement qualitative findings, research personnel additionally reviewed program records to ascertain data related to the *Reach* and *Adoption* of the ImPrEP Mexico demonstration project. The records review occurred in person at the implementation sites in Mexico City from June – August 2019.

### Measuring the REACH of ImPrEP México

The *Reach* of ImPrEP México among MSWs in Mexico City was determined by (1) the number of MSWs in Mexico City participating in ImPrEP México at the time of data collection and (2) the number of known MSWs in Mexico City who were eligible to participate in ImPrEP México at due to their HIV-negative status.

The number of MSWs in Mexico City currently participating in ImPrEP México was collected from existing project records at the two implementation sites in Mexico City: Clínica Especializada Condesa and Fundación México Vivo. The number of MSWs participating in ImPrEP México reflected the total number of participants enrolled in the program since the start of recruitment for ImPrEP México in February 2018 through the end of data collection for this study in August 2019.

The number of MSWs in Mexico City who are eligible to participate in ImPrEP México was estimated from *Punto Seguro* project records at Clínica Especializada Condesa (hereafter referred to as “Clínica Condesa”). *Punto Seguro* was an HIV/STI prevention program for MSWs at Clínica Condesa offered from May 2012 to May 2014 [5]. We used the number of MSWs who were HIV-negative at the end of *Punto Seguro* as an approximation for program eligibility because information on the number of HIV-negative MSWs in Mexico City are not currently available. *Punto Seguro* recruited MSWs from community sites where MSWs frequently work and from Clínica Condesa, and thus we believe it is a reasonable estimate of the number of HIV-negative MSWs in Mexico City [5].

### Measuring the ADOPTION of ImPrEP México

The *Adoption* of ImPrEP México was determined from existing ImPrEP México project data based on (1) the number of settings currently implementing the ImPrEP México protocol in Mexico City and (2) the number of settings in Mexico City eligible to implement the ImPrEP México protocol. Eligible settings were defined as those that were originally invited to implement the ImPrEP Mexico project regardless of whether or not they ultimately offered the program. Data from project records were validated with key informants of ImPrEP México during qualitative data collection.

### Analysis

We applied validated *RE-AIM* formulas to estimate ImPrEP Mexico’s *Reach* and *Adoption* [18]. *Reach* was calculated as the proportion of eligible participants (HIV-negative MSWs in Mexico City) who participated in the intervention (ImPrEP México in Mexico City). *Adoption* was calculated as the proportion of eligible settings (organizations or clinics in Mexico City originally invited to implement the ImPrEP México demonstration project) who implemented the intervention.

For the qualitative analysis, transcripts of the key informant interviews and focus group discussions were analyzed using the inductive, open coding approaches of grounded theory [22]. Key informant interviews and focus group discussions were analyzed together to bridge provider and patient perspectives on the barriers and facilitators to PrEP implementation in Mexico. Following best practices for conducting qualitative research using the *RE-AIM*, an initial coding scheme was developed by Hemant Kadiamada-Ibarra (HKI) (first author) and Nicola Hawley (NH) (co-author) based on the main questions asked and emergent themes from the data [23]. We then examined the qualitative data for emergent sub-themes. For the key informant interviews and the FGDs, three researchers (HKI, NH, and Roxana Rodríguez-Franco (RRF)) independently coded each transcript and then met to reach consensus on all final codes. Given the sample size, all content from all interviews and focus groups were coded. The initial coding scheme was refined during the coding process and edits to the coding scheme were applied to all transcripts. HKI entered the coded interviews into NVivo software (Version 12.0) to facilitate data management and analyses. A thematic analysis was conducted by HKI, NH, and RRF in which individual codes were read in aggregate and then used to create written summaries of each code.

## Results

### Reach

Sixty-eight male sex workers were enrolled and receiving PrEP through the ImPrEP México project: 35 through Clínica Condesa and 33 through Fundación México Vivo. *Punto Seguro* project records reported 707 HIV-negative MSWs in Mexico City. Thus, the 68 MSWs participating in ImPrEP México represent almost 10% of known HIV-negative MSWs in Mexico City.

### Adoption

Four sites in Mexico City were identified as eligible to implement the ImPrEP México protocol: Clínica Especializada Condesa (a government clinic), Clínica Condesa-Iztapalapa (a government clinic), Fundación México Vivo (a non-government organization) and Inspira Cambio (a non-government organization). However, only Clínica Condesa and Fundación México Vivo were actively implementing the ImPrEP México protocol at the time of this study. Inspira Cambio was not participating due to the closure of its primary facility in Mexico City for government use. No specific reasons were reported for Clínica Condesa-Iztapalapa’s lack of participation. Thus, half of the eligible settings in Mexico City were implementing the ImPrEP México protocol at the time of data collection.

#### Qualitative findings

Table 2 presents the key barriers and facilitators of PrEP uptake that emerged from the key informant interviews and focus group discussions. Example quotes that best capture each theme are presented in the main text with additional quotes representing the range of opinions provided in Supplementary Material 2. Findings relating to the *Reach* of PrEP among MSWs in Mexico City related to participant characteristics as well as barriers and facilitators to program *Reach*. Findings relating to the wider *Adoption* of the ImPrEP program in Mexico illuminated key barriers and facilitators to PrEP adoption.

**Table 2:** Themes impacting PrEP implementation and access among MSWs

Content Area	Respondent(s)	Emergent theme(s)
*RE-AIM Domain: REACH*		
Characteristics of ImPrEP Program participants	Key Informants	• Socioeconomic Status • Escorts vs. Street Sex Workers • Migration • Sexual Orientation
Barriers to *Reach*	Key Informants	• Lack of Current Strategies to Reach MSWs • Lack of Incentives for Participation
	MSWs	• HIV-related Stigma • Structural Barriers
	Key Informants and MSWs	• Lack of Awareness/Information about PrEP
Facilitators to *Reach*	Key Informants	• Connection to the Community • Online Presence
	MSWs	• Personalized, Humanistic Care
	Key Informants and MSWs	• Organic Transmission (Word of Mouth) • In-person Interaction and Outreach
*RE-AIM Domain: ADOPTION*		
Barriers to *Adoption*	Key Informants	• Lack of Public Policy • PrEP-, HIV-, and Sexual Minority-related Stigma • Limitations of ImPrEP México
Facilitators to *Adoption*	Key Informants	• NGOs • Sensitized Providers • Existing Infrastructure at the National Level and Clinic Level
CROSS-CUTTING THEMES FOR *REACH* AND *ADOPTION*		
Demand for PrEP	Key Informants and MSWs	• High Patient Demand

*PrEP* Pre-Exposure Prophylaxis, *MSWs* Male Sex Workers, *NGOs* Non-governmental Organization

*Notes:* Key informants participated in in-depth interviews. MSWs participated in focus group discussions. Key informants and MSWs were consulted regarding PrEP *Reac*h among MSWs in Mexico City. Solely key informants were consulted regarding PrEP *Adoption* in Mexico

### Participant characteristics

Key informants reported that MSWs receiving PrEP through the ImPrEP México project were mainly online escorts (i.e., sex workers who solicit clients through websites or applications), of medium to medium-high socioeconomic status, and migrants from South America. MSWs who were least likely to be reached by the ImPrEP project included street sex workers (i.e., those who seek clients in public spaces in Mexico City), those of low socioeconomic status, and migrants from states within Mexico. Key informants further reported that MSWs who self-identified as heterosexual, rather than homosexual, were less likely to be engaged in ImPrEP. The following quote encapsulates these ideas:

*So, those who are being recruited are those who do their work and advertise on social networks, through applications. But, the one that is not [being reached] is the one in the specific places where you go, and you know that there is male sex work. So, that is a profile that has a low socioeconomic status and that has sexual relations with women. They probably have a partner, and they came from another state of the country to look for a better opportunity, but they did not find it. So, it is very common that what they do is sex work. So, there are certain zones where they pursue an income from that sex work. They are not gay; they are not homosexual* *.*

Key Informant Interview #6

### Barriers to reach

When asked about the barriers to reaching MSWs in Mexico City, key informants admitted that ImPrEP México has not focused on developing strategies to reach MSWs; rather, the program’s focus has been on MSM and transgender women.

*In reality, we have not made a very wide dissemination in Mexico City. The strategy of communication or dissemination was, let’s say, between people who knew each other, through word of mouth … that was the main thing. And then, there were a few months that we put announcements on digital information platforms … on Facebook, on social networks, but it was a very limited time. And I currently believe those who are coming are a very small group...we have not covered the whole population of MSWs in Mexico. We still need to do a lot of work. […*] *The strategies we used were directed in general to MSM and trans women. We only focused on those groups, but we did nothing specific for MSWs. So, what we have, on the one hand, is the people who come to the clinic regularly to get tested for HIV, and we have not been able to measure how many found out by other means. We are well below achieving a wide coverage in Mexico City.*

Key Informant Interview #2

Key informants attributed the low *Reach* of PrEP among MSWs to there being no incentives for MSWs to initiate and adhere to PrEP. A lack of incentive was considered particularly problematic for MSWs of lower socio-economic status whose need to maintain employment through street sex work often meant that seeking PrEP and HIV services would mean a loss of clients and income. Key informants from both clinical and community-based settings felt incentives are a critical tool for increasing MSWs’ care engagement and medication adherence.

In comparison, FGD participants repeatedly emphasized HIV-related stigma as a prominent barrier to accessing PrEP and HIV health services. This stigma was felt at the individual level, when participants’ own fears of knowing their HIV status prevented them from seeking preventive services, and at the societal level, when participants worried about potential stigma that they would face from community members and other MSWs if they were seen receiving HIV-related services. MSWs also described geographic distance and clinic wait times as barriers to accessing PrEP and HIV prevention services.

*The insecurity and lack of information that many people have [is a main obstacle for people to use HIV prevention services]. Look, I’m from a pueblo [small town], so many people who are positive sometimes don’t even know that they are positive. But, they already have an idea that they are positive, but they are not capable [to seek help], due to insecurity of how their treatment will go. In some states of the country, they think that HIV treatment is very expensive; they don’t even know that it is free. I am from the state of Tabasco. I brought a friend who was positive [to Mexico City]. I took him to Clínica Condesa. Unfortunately, they told him that he had to do the treatment in the state of Tabasco where there, if they find out you are positive, you will be judged harshly...and so my friend passed away in November, because he was not given the medicine.*

FGD Participant #3

Both key informants and FGD participants agreed that lack of awareness of PrEP is a major barrier to PrEP *Reach* among MSWs. Only 3 of 8 FGD participants said that they had heard of PrEP, and most said that they only used condoms as a means of preventing HIV. MSWs also shared that there is a high amount of misinformation among MSWs regarding what PrEP is and the cost of obtaining PrEP in Mexico. Key informants were also keenly aware of that most MSWs were not aware that PrEP can be accessed free of charge and that treatment involves more than just taking a daily pill.

### Facilitators to reach

Key informants described “Connection to the Community” as one of the most important facilitators of program *Reach*. Respondents said that Clínica Condesa and NGOs’ connections to MSWs and other communities of sexual minorities in Mexico City (mainly MSM and transgender populations) facilitated in-person communication about ImPrEP México during patients’ regular visits for HIV- and STI-related care. ImPrEP México’s online presence (through Facebook and the social media campaign “HablemosdePrEP”) may have helped MSWs learn about PrEP.

*The most effective is that the people who come to get tested at the Clínica Condesa... the MSWs who come to have their HIV tests regularly at the clinic...are identified and immediately informed of PrEP. The vast majority, at the time you invite them in-person, agree to participate because if they read on social media or read on the internet, they have a harder time making the decision to come. But, if they are getting an HIV test and you talk to them about the program, they immediately connect themselves to the program.*

Key Informant Interview #2

Another key facilitator identified by key informants and FGD participants was interpersonal communication about ImPrEP México between sex workers. Key informants emphasized that organic transmission of information about PrEP between MSWs facilitated recruitment and program participation. FGD participants mentioned that it was through word-of-mouth that they were able to encourage their friends who are also sex workers to seek and access HIV prevention services. Both key informants and FGD participants positively recognized the in-person outreach done by Clínica Condesa and NGO staff in “zonas” of Mexico City where street sex work happens. However, MSWs noted that taking time to talk to outreach workers can represent a missed opportunity cost of soliciting a potential client, and several said they were uncomfortable speaking with outreach workers in public spaces. Most FGD participants preferred private and personalized encounters for PrEP education and engagement, similar to the focus groups conducted for this study. Several also stated that transportation assistance was important for accessing PrEP services.

*“[...] before I used to work on the street. Now, it has been more or less a year that I have gone out to the street, but they used to visit us from different associations and give us small talks there on the street. I thought it was super cool, but sometimes...well, we can’t be paying so much attention to them because either we attend to them or the client leaves us. [...] So, I do think it’s really cool, but it’s not exactly the right place to do it. [ …] my ideal I think would be to look for us, contact us and bring us [to the facility], just like now.”*

FGD Participant #2

### Barriers to adoption

Key informants reported that the lack of state- and national- level policies for PrEP distribution contributed to higher medication costs and a lack of educational resources for patients and providers. Key stakeholders also acknowledged that many providers in Mexico would not willingly distribute PrEP because doing so would require them to engage with marginalized communities living with stigmatizing health issues including HIV and STIs.

*I believe that PrEP is a strategy that, since it arrived in the country, has a stigma component that implies that: if you bring PrEP, you will encourage people have sex without a condom. So, I believe that civil organizations and institutions that are closer to the latest evidence regarding HIV prevention may be more willing to implement it than some other more conservative services, which probably would not. Also, the population of men who have sex with other men is stigmatized for being gay, unlike men who are heterosexual. Then, there is that stigma of HIV, and the stigma that we are promiscuous. So, I think there are still civil organizations in Mexico that say, “Why bring PrEP so they have sex without a condom if we still have people living with HIV who have no treatment?” [...] But there are others like us that say, “Well, if we do not distribute it, the person will go looking for it and buy it in the black market and that can have repercussions in the long run: acquiring HIV, acquiring an STI, generating resistance, etc.”*

Key Informant Interview #6

The rigidity of the ImPrEP México protocol – in terms of appointment scheduling, maximum number of participants and MSM/transgender eligibility criteria – was an additional barrier to wider adoption of the protocol in the country.

*Clínica Condesa, I tell you, is very user-centered, and we try to do a different type of intervention... a differentiated attention for each person. In contrast, the ImPrEP protocol is the same for everyone and is very rigid. So, initially, it cost us a lot of work. [...] Now, to scale it up, it has to be more flexible. If you do not make it more flexible, there is no way to take it to a larger scale because you need more staff; you need other resources.*

Key Informant Interview #2

### Facilitators to adoption

To facilitate ongoing and future adoption of PrEP, key informants from both clinical and community sites emphasized the important role that NGOs in Mexico play in delivering HIV and STI-related health services to at-risk populations. NGOs are able to reach marginalized populations in the community, have shorter wait times compared to public sector clinics and have a longstanding leadership role in HIV prevention in the country.

*Organizations of the civil society have always possessed an abundance and richness in their experience. The issue of HIV in Mexico, along with the issue of HIV diagnosis, has always been spearheaded by organizations of the civil society. They are the ones who always took that fight and always kept pushing forward. [...] They know the needs of the community … of MSWs, of MSM. They have been involved in the realities of the population. So, what was done is that they were added to the intervention, and I think there are more organizations of the civil society that can offer PrEP. Surely, there are already organizations of the civil society that are offering PrEP outside of the ImPrEP protocol because of that same experience. If they were able to give antiretroviral treatment to people living with HIV when they could not get it in health services in the past, then I have no doubt that they are already doing it with PrEP in some way. So, I think that these organizations can distribute PrEP.*

Key Informant Interview #7

Other facilitators to wider adoption of the ImPrEP Project included engaging providers who are sensitized to working with MSM, transgender and sex worker populations, and building on existing infrastructure to increase PrEP distribution and scale-up. Building on existing infrastructure would include implementing ImPrEP Project within NGOs that already address HIV. At the clinic-level, existing infrastructure that was seen to be useful for future scale up included sensitized healthcare staff, a team-based approach to delivering PrEP, and shorter wait times.

### Demand for PrEP

Both key informants and FGD participants highlighted the pervasive demand for PrEP among MSWs in Mexico. At the clinic level, there is a wait list of eligible individuals wanting to enroll in ImPrEP México, as well as phone calls from individuals living outside of Mexico City. Key informants additionally reported not encountering any eligible participants who declined to enroll in ImPrEP.

*We have even had calls from other states. We have been called [by the general population] from Cancún, the State of Mexico, and Hermosillo with the interest of acquiring PrEP. […] We have a lot of demand from all users. In fact, we have a waiting list, but only people who were able to join and register last year are being followed up. Right now, we are waiting for the coordinators to give us the green light to continue. We will probably start recruiting more people soon because there were some changes in the project.*

Key Informant Interview #6

After receiving information about the ImPrEP Project and how to access PrEP in Mexico, 6 out of 8 MSWs expressed high interest in enrolling.

## Discussion

This study used the *RE-AIM* framework to evaluate the *Reach* and *Adoption* of the ImPrEP México demonstration project among male sex workers in Mexico City. Findings from this analysis indicate that PrEP has a low *Reach* (10%) among male sex workers in Mexico City, especially those of lower socioeconomic status. Only half of the eligible settings in Mexico City were actively implementing the ImPrEP México protocol.

The low *Reach* of ImPrEP México among MSWs in Mexico City can be attributed, in part, to the lack of recruitment strategies targeted to MSWs and minimal PrEP awareness among this population. Lack of PrEP education is a well-documented barrier to PrEP uptake among sex workers in low- and middle-income countries. Restar et al. reported low awareness and knowledge of PrEP among female and male sex workers in Mombasa, Kenya who solicited clients in bars and nightclubs [24]. Edeza et al. also reported limited prior knowledge of PrEP among MSWs in Mexico City; the majority of participants in this sample were MSWs who only solicited clients on the street [6]. Despite low awareness of PrEP among sex workers, both studies reported high interest and willingness among sex workers to use the medication once they were informed, a theme that was also common among the MSWs in our study. Low *Reach* among MSWs in Mexico City can also be attributed to HIV-related stigma. While the deleterious effects of HIV-related stigma on mental health outcomes of HIV-positive individuals has been well documented [25–28], the present research illuminates how HIV-related stigma can also prevent HIV-negative individuals from seeking care and prevention services [28].

The current study builds on prior research which found that compared to internet escorts, MSWs who engage in street sex work have higher rates of HIV risk behaviors but are less likely to be engaged in PrEP services [29–34]. Yet there is a lack of evidence as to *why* HIV prevention programs are not as effective at engaging street-based MSWs. Our findings suggest that barriers to widespread PrEP uptake are related to challenges of poverty, lack of information and stigma faced by this subgroup of MSWs [6]. Structural barriers (transportation costs and social stigma), individual-level barriers (drug use, mental stability, and fear) and unsatisfactory healthcare consultations have shown to prevent female street-based sex workers from accessing health and social services in both high and low-income countries [35, 36]. In these settings, in-person outreach and follow-up from peer educators improved linkage to HIV care among female sex workers, a strategy which holds promise for engaging male sex workers in PrEP services [37, 38].

A key finding of this study was the importance of offering incentives to increase PrEP engagement, especially in the context of street sex workers where the opportunity cost of lost business is a major disincentive. The use of CEIs during the *Punto Seguro* randomized trial among MSWs, mainly street sex workers, in Mexico improved the frequency of clinic visits by 10–13 percentage points and condom use 10–15 percentage points relative to controls [5]. CEIs have shown to be especially impactful for improving health behaviors among high risk populations, such that extending CEIs for PrEP adherence could prevent HIV acquisition in MSWs who are street sex workers. For others, for example the higher-income escorts, cash incentives may not be as effective; further research should focus on how best to incentivize this segment of the MSW population.

For *Adoption*, our study reported 2 out of 4 eligible settings adopting the ImPrEP México project in Mexico City. *Adoption* of the ImPrEP project is happening in settings that have sensitized providers, exceptional leadership in HIV/AIDS care, and connections to at-risk communities. These characteristics make these organizations well-suited to offer PrEP to at-risk populations in Mexico City, in comparison to other healthcare settings where stigma toward sexual and gender minorities is common [39]. Settings that were not selected for participation did not actively refuse, but rather were considered not to have adequate infrastructure/patient flow in place to support *Adoption*. A key takeaway from this analysis is that future efforts to scale-up PrEP in Mexico should build upon existing clinical and community-based institutions where MSWs already feel comfortable receiving HIV/AIDS prevention and treatment services. This aligns with perspectives from a multinational study of 35 PrEP policymakers and providers in LMICs, all of whom support the delivery of PrEP programs via established healthcare facilities and non-governmental organizations [40]. Several other studies have highlighted the important role that local NGOs play in the HIV/AIDS sector, due to these organizations’ capacity to mobilize communities and resources, reach high risk populations, and provide HIV-prevention services to marginalized groups who may not be able to access traditional clinic settings [41, 42]. In Mexico, NGOs and the creation of the AIDS National Prevention and Control Council (CONASIDA) were integral to curbing the AIDS epidemic [43]. Building on this longstanding and concerted effort of non-governmental institutions – using approaches that address issues of stigma and promote greater community outreach - may support a wider adoption and uptake of PrEP services in the country.

### Strengths and limitations

A key strength of this study is the inclusion of qualitative data from both program implementers and program participants. Perspectives of MSWs are noticeably sparse in the HIV literature; this study adds to the paucity of data surrounding this hard to reach population. Additionally, this is the first time that implementation science research is being conducted in the context of HIV prevention in Mexico City. Furthermore, the current study highlights specific ways in which public health programs can increase the *Reach* and *Adoption* of the ImPrEP demonstration project, which will be critical if or when the project becomes a permanent public health initiative.

This research is not without limitations. First, there are limitations in the estimation of HIV-negative MSWs in Mexico City. This study’s estimation cannot be interpreted as an exact number of HIV-negative MSWs in Mexico City; to-this-date, no research has quantified that number. Given that MSWs are a highly stigmatized population and few MSWs openly disclose their occupation as sex workers, it is difficult to quantify exact numbers of MSWs [5]. For this reason, the best way to approximate these numbers is by using data from clinics that MSWs in Mexico City frequently seek services from. Clínica Condesa, the site of the *Punto Seguro* project, has seen *n* > 1100 MSWs at least once for STI testing and treatment or other services and has become a regional reference center for providing free services in a respectful and non-discriminatory environment [5]. This is why the estimate of HIV-negative MSWs in Mexico City from *Punto Seguro* data is still valuable to use for our measurement of *Reach*.

Secondly, the number of HIV-negative MSWs in Mexico City is most likely underestimated. *Punto Seguro* solely recruited street-based sex workers in Mexico City; thus, MSWs who engaged in internet-based sex work were excluded from the number of HIV-negative MSWs in Mexico City, which implies that our estimates of program *Reach* are less than presented. It also implies that the enrolled population is likely unrepresentative of the larger community. We were unable, however, to quantify the degree to which this was true, and therefore could not accurately address the “representativeness” aspect of *Reach*.

Lastly, participants in the FGDs were recruited from public venues where project staff conduct outreach. MSWs who are solely online escorts were not invited to participate, which limits the generalizability of study findings to the entire male sex worker population in Mexico City.

## Conclusion

MSWs in Mexico City are at an increased risk of HIV infection, yet have low engagement in HIV prevention services. To increase the *Reach* of PrEP offerings among MSWs in Mexico, structural interventions must be able to address the structural and stigma-related barriers faced by the most socio-economically disadvantaged MSWs. The ImPrEP demonstration project alone will not be able to meet the growing demand for PrEP in the country. Scaling up PrEP programs in Mexico will rely on the existing non-governmental organizations and public health institutions that already serve HIV-affected populations and the creation of public policy for permanent and accessible PrEP services.

## Supplementary Information

**Additional file 1: Supplementary Material 1.** Semi-Structured Interview Guide for Key Informant Interviews and Focus Group Discussions. English language version of the semi-structured interview guide that was used to conduct in-depth interviews with key informants and focus group discussions with male sex workers during qualitative data collection in Mexico.

**Additional file 2: Supplementary Material 2.** Additional Quotes for REACH and ADOPTION. Additional example quotes related to key barriers and facilitators of PrEP uptake that emerged from in-depth interviews with key informants and focus group discussions with male sex workers during qualitative data collection in Mexico.

## Data Availability

All data generated or analyzed during this study are included in this published article. The datasets used and/or analyzed during the current study are available from the corresponding author on reasonable request.

## Abbreviations

AIDS: Acquired immunodeficiency syndrome
FGD: Focus group discussion
HIV: Human immunodeficiency virus
MSM: Men who have sex with men
MSWs: Male sex workers
NGO: Non-governmental organization
PrEP: Pre-exposure prophylaxis
