# Publisher Correction To: Barriers and facilitators to pre-exposure prophylaxis uptake among male sex workers in Mexico: an application of the *RE-AIM* framework

**DOI:** 10.1186/s12889-021-12415-y

**Published:** 2022-01-27

**Authors:** Hemant Kadiamada-Ibarra, Nicola L. Hawley, Sandra G. Sosa-Rubí, Marta Wilson-Barthes, Roxana Rodríguez Franco, Omar Galárraga

**Affiliations:** 1 Brown University School of Public Health, Providence, RI 02912 USA; 2 Department of Chronic Disease Epidemiology, Yale School of Public Health, New Haven, CT 06510 USA; 3 Division of Health Economics, National Institute of Public Health (INSP), 62100 Cuernavaca, CP Mexico; 4 Department of Epidemiology, Brown University School of Public Health, Providence, RI 02912 USA; 5 Center for Demographic, Urban, and Environmental Studies (CEDUA), The College of Mexico, 14110 Mexico City, CP Mexico; 6 Department of Health Services, Policy, and Practice, Brown University School of Public Health, Providence, RI 02912 USA

**Correction to: BMC Public Health 21, 2174 (2021)**

**https://doi.org/10.1186/s12889-021-12167-9**

During the publication of the original article [1] several changes were not correctly implemented. These changes are as followed:

1. The order of the author list was not correct
  - The incorrect author order was: Hemant Kadiamada-Ibarra, Nicola L. Hawley, Sandra G. Sosa-Rubí, Marta Wilson-Barthes, Omar Galárraga and Roxana Rodríguez Franco
  - The correct author order is: Hemant Kadiamada-Ibarra, Nicola L. Hawley, Sandra G. Sosa-Rubí, Marta Wilson-Barthes, Roxana Rodríguez Franco and Omar Galárraga
2. Several smaller formatting changes for the quotes

The original article has been updated to rectify these errors.

The publisher apologizes for the inconvenience caused to the authors & readers.
