# Supplementary material for: Barriers and facilitators to pre-exposure prophylaxis uptake among male sex workers in Mexico: an application of the RE-AIM framework
Source: BMC Public Health. 2021 Nov 27;21:2174. doi: 10.1186/s12889-021-12167-9 (PMC8626882; doi:10.1186/s12889-021-12167-9)
Supplement: Supplementary file 1 — Additional file 1: Supplementary Material 1. Semi-Structured Interview Guide for Key Informant Interviews and Focus Group Discussions. English language version of the semi-structured interview guide that was used to conduct in-depth interviews with key informants and focus group discussions with male sex workers during qualitative data collection in Mexico. [file 12889_2021_12167_MOESM1_ESM.pdf]

Supplementary Material 1. Semi-Structured Interview Guide for Key Informant Interviews and Focus Group Discussions

Key Informant Interview Guide	Focus Group Interview Guide
Questions about *Reach*	Questions about *Reach*
1. What are the common characteristics among the MSWs starting PrEP through ImPrEP? Are some MSWs more open to the idea of starting PrEP than others?<br>2. Are there common characteristics among the MSWs who are not participating in ImPrEP?<br>3. What are some of the reasons that MSWs decline to participate in ImPrEP?<br>4. Who are the types of MSWs that ImPrEP is not reaching?<br>5. What methods have you found to be effective for reaching these people so that they start PrEP?<br>6. How do MSWs find out about the ImPrEP program?<br>7. Are there ways that MSWs could be better reached in Mexico City to start PrEP?	ACCESS TO HEALTHCARE SERVICES<br>1. When you need medical care, where do you go?<br>2. What types of health services are the ones that you demand the most?<br>3. Have you received any type of service related to HIV prevention? How was the experience? What did you like? What did you not like?<br>4. How would you like care in prevention services to be? (how the staff treat you, hours, location, etc.)<br>5. What do you think is the main obstacle to seek prevention services related to HIV?<br><br>PrEP<br>1. Have you heard about PrEP? What have you heard? What advantages and disadvantages does it have? Where was it that you obtained this information? (Then, provide general information about what PrEP is and specifics about how to take it, who can take it, and where to get it)<br>2. If you already live with HIV, what is your opinion about PrEP? What is your opinion about other STIs?<br>3. Who should receive information about PrEP? How should this information be presented/be disseminated (places, strategies (print-outs, videos, etc.)?<br>4. Are you willing get screened for PrEP? (keeping in mind that it is free of charge)

	5. What would happen if PrEP were available for everybody to use? (explore what would happen with condom use, transmission of other STI, enjoyment of sexual life)<br>6. Where would you feel most comfortable getting PrEP?<br>7. If you decide to use PrEP, with who would you and with who would you not share this information?<br>8. What do you think are the main obstacles that would prevent you from taking PrEP?
Questions about *Adoption*	
1. What types of settings were chosen to distribute PrEP?<br>2. Which settings were excluded from ImPrEP? Why?<br>3. In Mexico City, are some places more likely to distribute PrEP than others?<br>4. What are the characteristics of the providers who received training to distribute PrEP?<br>5. Are certain providers more likely to receive training for PrEP than others? What are the characteristics of the providers that aren’t distributing PrEP?<br>6. How well is [the setting] adopting the procedures of ImPrEP?<br>7. In the future, how feasible is it for more institutions to adopt PrEP? Are there any medical clinics or practices that would not distribute PrEP?	
