# Supplementary material for: Barriers and facilitators to pre-exposure prophylaxis uptake among male sex workers in Mexico: an application of the RE-AIM framework
Source: BMC Public Health. 2021 Nov 27;21:2174. doi: 10.1186/s12889-021-12167-9 (PMC8626882; doi:10.1186/s12889-021-12167-9)
Supplement: Supplementary file 2 — Additional file 2: Supplementary Material 2. Additional Quotes for REACH and ADOPTION. Additional example quotes related to key barriers and facilitators of PrEP uptake that emerged from in-depth interviews with key informants and focus group discussions with male sex workers during qualitative data collection in Mexico. [file 12889_2021_12167_MOESM2_ESM.pdf]

**Supplementary Material 2: Additional Quotes for *REACH* and *ADOPTION***	
RE-AIM Domain: REACH	
Content Area	Quotes
Characteristics of ImPrEP Program participants	Socioeconomic Status<br><br>*I think that in general the profile of people who have access to PrEP, not only among men who have sex with other men, does speak to a socioeconomic level of middle and upper class. So, this type of population is a population that has access to a cell phone with data. Then, from there they also have access to a lot of information about the latest developments in HIV prevention technology. [...] So, the profile of male sex workers that we currently have is in this block of people with privileges and these advantages. And the other group is people who do sex work directly on the streets. They have less access to information, they have less access to health services, and so they don't know what PrEP is. And the little they do know is probably because someone told them…because they read something, but they don't have all the information or complete idea of what it is, how it works, and where they can get it.*<br>Key Informant Interview #6<br><br>Escorts vs. Street Sex Workers<br><br>*There are sex workers from an area of the district of Mexico City called Alameda. There, there is a population that is in a situation of living on the street, of abandonment, and of substance use. They are not structured; they do not have an attachment to the clinic or to treatment for other projects we have had. So, these [MSWs]...this project has not reached them because they are so much more difficult to get them to come to the clinic and have care provided to them. If any of them could be in the program, it would be great. I think they will be, but it will be more complicated for them to open up to a protocol of follow-up as specific as is requested here.*<br>Key Informant Interview #3<br><br>Migration<br><br>*And another population within this large universe, I think we have foreigners from Colombia and Venezuela. They are taking PrEP through ImPrEP México and they do have a rather peculiar requirement that goes beyond the parameters of sex workers here in Mexico ... I don't know if it's the culture or that they feel that everything is easier for them, but they do have more of a demand for time, for services, and yes, they get desperate for time if they are not satisfied according to their wants.*

	Key Informant Interview #3<br><br>*A large part [of the MSWs in ImPrEP Mexico] are South Americans…they are South American immigrants with no legal status in the country.*<br>Key Informant Interview #8<br>*Exactly, the characteristic of their immigration status corners them a bit to exchange sexual acts for some economic or material good.*<br>Key Informant Interview #9<br><br><u>Sexual Orientation</u><br><br>*I cannot speak exactly of that group [of MSWs in ImPrEP México] because I have not participated in that analysis of the men who do sex work that take PrEP with us. But, we do know the target population—the one which we attend to. We know that they are mostly young guys. We have tried to target the population between 18 and 25 years who perform sex work. They are guys of very low resources. More often than not, they are migrants. They are from other states in the country and come to the city [Mexico City] to look for employment. And very often, they perform sex work from a very precarious economic situation. And often, they are boys who do not identify as homosexual and have female partners...well, their sexual orientation is heterosexual. But in their work, their clients are men—which makes them a bridge population of HIV. That is to say that this connects the epidemic to women and children...there are cases of vertical transmission. So, there are 2 different epidemics: that of women, or heterosexual, and that of men who have sex with men, which are connected by this bridge population that are male sex workers with a heterosexual orientation or a heterosexual preference but who have work with male clients.*<br>Key Informant Interview #4<br><br>*I think that in general the profile of people who have access to PrEP, not only among men who have sex with other men, does speak to a socioeconomic level of middle and upper class. So, this type of population is a population that has access to a cell phone with data. Then, from there they also have access to a lot of information about the latest developments in HIV prevention technology. [...] So, the profile of male sex workers that we currently have is in this block of people with privileges and these advantages. And the other group is people who do sex work directly on the streets. They have less access to information, they have less access to health services, and so they don't know what PrEP is. And the little they do know is probably because someone told them…because they read something, but they don't have all the information or complete idea of what it is, how it works, and where they can get it.*

	Key Informant Interview #6
Barriers to *Reach*	Lack of Current Strategies to Reach MSWs<br><br>*Well, I think that they are sex workers who are less-favored, with a low/medium-low socioeconomic class. I think that they are the ones that until now have not been able to be reached so that they can also benefit from this intervention. Since we are not doing direct work in the field and we are waiting for them to come to us, it is very difficult for them to reach the clinic by their own means and merits. I think that this population is the one that we are not reaching because of the same peculiarity of this project and the population as well.*<br>Key Informant Interview #3<br><br>Lack of Incentives for Participation<br><br>*We must find a mechanism for them to come to the clinic, find a mechanism for them to adhere to the treatment because the treatment is not just taking the pill. It is a medical treatment that involves going to the clinic, laboratories, etc. And in the less favorable population, apart from the medication, we must look for another incentive that makes them come to the clinic because we often do not a way to contact them because they do not have a cell phone, they change location a lot, and they do not adhere much to a health center.*<br>Key Informant Interview #3<br><br>*There are many studies on how to incorporate MSWs into health services, and the most successful involve giving them incentives, even money. We do not give the resource that pays them to come to their appointments, but there are studies that pay them to come or give them support for transportation. They are the most successful. And it is logical because the time of a sex worker is money and time that is not with a client is wasting money.*<br>Key Informant Interview #8<br><br>*I've noticed when I talk to people that they know that there is a place that gives PrEP, but it takes time to make the decision and go. And more to the MSWs group because they think in terms of that they are wasting time and losing income. They think, "I'm going to the clinic, I'm going to be there for two hours, three hours, and that means I'm not going to have an income or I'm going to lose a client."*<br>Key Informant Interview #2<br><br>HIV-related Stigma<br><br>*No, well, it [HIV] scares me. It scares me a lot. It's been two years since I*

	took a test. I had nothing, but from those two years to now, I don't know…<br>FGD Participant #6<br><br>*It cost me a lot of work [to get tested for HIV]. The most difficult thing is to take the test when you already know what you could have it...because of fear. But, it is also a matter of people, because there are many people, for example, from urban areas that are very closed and will not go to the clinic to do an HIV test out of fear.*<br>FGD Participant #3<br><br>*It's more, even [the fear of] what they will say...the fear that they see you outside of clinical Condesa. That information spreads and more if you are well-known, right? The thousands of times that you happen to be outside of Clínica Condesa everyone will know. So, when you enter or stand at the door, you are going to be afraid...you enter into panic when you get your results and even if you are negative, they already saw you at Clínica Condesa. And, well, what are they going to say, right? Although you only went to peek inside…*<br>FGD Participant #2<br><br>*P6: I saw in the Alameda about a month, a month and a half ago that they were doing free tests…*<br>*M: And what did you think of that?*<br>*P6: Well that's fine, they support people, right? To take away the fear a bit. In fact, I said: "I should go to do the test…*<br>*M: ...and then?*<br>*P6: No, no, I didn't.*<br>*M: Why?*<br>*P6: Because I was a coward.*<br>*M: Were you scared?*<br>*P6: The truth is, yes.*<br>FGD Participant #6 & Moderator<br><br>*M: Ok, and when you have received some type of prevention service, have they told you: "Hey, use a condom." Have you had that experience?*<br>*P7: Yes, [...]there in the Alameda, as the others have said they [Clínica Condesa staff] go to Alameda...I didn't really approach them though. I only saw them.*<br>*M: Why didn't you decide to go? Did you feel worried…?*<br>*P7: Yes...*<br>*M: That they would see?*<br>*P7: Yes.*<br>FGD Participant #7 & Moderator<br><br><u>Structural Barriers</u>

M: And well [...] for those of you who are here and who want to go to the clinic [Clínica Condesa] to get tested, you can go every day during morning hours.
P5: But do I have to go all the way to the clinic?
M: You can go to one in Iztapalapa too. There are two clinics. Well, what we are seeing is that it [HIV testing] is free, right? And, the Juanacatlán metro is here.
P5: Yes, but right now I don't even have a 'peso' [Mexican currency]...
M: Not even for the metro?
P5: Not even for a metro ticket.

FGD Participant #5 & Moderator

M: So, you're telling me that people are going to prefer taking PrEP to using a condom?
P3: No, I'm thinking the opposite...they prefer condoms because it's easier to get it, faster and you don't have to go to a clinic.

FGD Participant #3 & Moderator

If right now it [PrEP] is just starting, I don't think they'll sell it or give it to you anywhere, right? I return to the same thing...the people who live in the State of Mexico or who live in rural areas...why the hell do they have to come all the way down by donkey to some association here? That seems to be an impediment to me, right? In general, in Tepotzotlán, in the State of Mexico, [...] there are also sex workers, right? There are people who want that service...just because they are from the ranch does not mean that they will not 'coger' [in English: to have sex]. There are even more people who want it there too.

FGD Participant #5

Lack of Awareness/Information about PrEP

Nothing has been done with regard to education. Well I say no education because the project has not had any intervention. That is to say that people cannot access PrEP if they do not know that it is available. And also, for them to access PrEP, they have to know what PrEP is. Until that step is taken, they are limited. That is to say that all civil organizations or all health spaces that serve key populations or where they are young people should have access to information, so people are able to say "I want PrEP. I want to enter the PrEP project." If not, the person who has access is the user who regularly goes to civil organizations that are within the project…where we tell them, "There is also a PrEP project." Here in Inspira, you come and there is the PrEP poster. [...] We need to tell people that there is a highly effective HIV prevention strategy, more effective than condom use and that people can access it.

Key Informant Interview #6

	The vast majority of the answers...when we ask, "How did you find out about PrEP?" ...the vast majority tell you that they learned about it through someone they know who uses PrEP. Most have read something on social media about PrEP, but by reading it on social media, they do not get a very good idea of what PrEP is. When they ask their friends who already use it, then they become clear about the method and where they can get it. Although, it is also important to mention that for the vast majority of young people, the strategy of PrEP is being reduced to only using the medication. The other components of PrEP have been lost sight of. The other components of PrEP are having regular HIV tests, continuing to use prevention methods, methods to not expose yourself to HIV and other sexually transmitted infections, and doing check-ups on your health conditions.<br>Key Informant Interview #2<br><br>*M: So, in this block, we are going to talk about PrEP. Has anyone heard about what PrEP is?*<br>*P6: No, not until now right now.*<br>*P4: No idea.*<br>*P5: No, I haven't heard…*<br>*M: Not even through some gossip that someone told you?*<br>*P7: No, neither do I, not until now…*<br>FGD Participant #4, #5, #6, #7 & Moderator<br><br>*I have heard very little. In reality, there is no information that I have seen on the internet or through advertising. It wasn't until I went to Clínica Condesa and saw there that it said 'PrEP'. What I have heard has been from acquaintances or from people who have told me that they take it, and the little I know from my ignorance is that it is to prevent the HIV virus. I know it's an experiment...well, I don't know if it is exactly an experiment, no? I know that there's like an agreement, right? It's just starting, right?*<br>FGD Participant #1<br><br>*In fact, I have friends who take PrEP [...] more awareness is needed, because many think that it is already a treatment for someone who is already positive [...]. There is a huge confusion.*<br>FGD Participant #2
Facilitators to *Reach*	<u>Connection to the Community</u><br><br>*The first is the advantage that we have because we already had a Punto Seguro project that was directed towards male sex workers. So, this advantage was what facilitated things. Some of them [MSWs] already*

	knew us. They were coming for their follow-up for other protocols or projects, and they got to know the clinic, the staff, and the service. So, that facilitated the opportunity where some of us could say: "Now there is the ImPrEP Mexico project. There is a PrEP Seguro project." They trust the information we have given them, and this has also facilitated things.<br>Key Informant Interview #7<br><br>*Really in those cases, what worked there was when people came for a rapid HIV test and you would detect those aspects that show that the person is at risk of acquiring HIV. And that was exactly where you noticed in what area he lived and what he did for a living. Sometimes, they are people who do not leave the 'colonia' [in English: neighborhood] in which they live. So, that's where we would talk about PrEP and talk about the service that existed and how they could access and how they could add themselves to the list. So, it would be better [to recruit] more during the part of detection day to day because we do understand that in the HIV part, there is much of that population of limited resources and of poverty—they are more vulnerable by socioeconomic aspects.*<br>Key Informant Interview #5<br><br>*Currently, I can tell you that they are the two most successful are by word of mouth and by now coming to the clinic on a regular basis because few have come because of what they saw on Facebook or on social media. Generally, those who came were because they had already been tested for HIV here at the clinic.*<br>Key Informant Interview #2<br><br>*Well, I think that one has been that, by coming to take the test directly and identifying them as candidates and explaining to them what it is about and why they are at constant risk…that had been a method that has been effective. Another that happens a lot is that many perceive themselves as subjects at risk and they come requesting this possibility.*<br>Key Informant Interview #3<br><br>*And the other way they learn about ImPrEP is when they come here for follow-up...that is, their routine HIV or syphilis exams. Well, they come and may see some small announcement that we have there in the hallway, or when the counselor is delivering the results of their exams, they may say, "Maybe you can add this PrEP option to what you are doing for prevention." In total, it has been different mechanism that have been part of the promotion."*<br>Key Informant Interview #7<br><br>Online Presence

Well, one way is through the social media of the clinic. We have a Facebook page for the area of counseling. So when the protocol began, we posted: "You can make an initial appointment to see if you are a candidate." Another is an official ImPrEP communication page in Mexico called "HablemosdePrEP.com". There are videos and information that identify which populations are candidates. Highly popular digital platforms in Mexico have interviewed key people—people involved in the project or people who serve as an image, and that also attracts the population.

Key Informant Interview #3

And digitally we have three strategies. One is through social networks. We have a Facebook site called "Hablemos de PrEP". All the guidelines that we talk about for PrEP are very sectorized to all the populations we are looking for. That is one. Another strategy is virtual approaches: counselors have a profile on networks…on Grindr, on Scruff, on ManHunt. They look through the profiles that are there and invite them to come and take PrEP. This is another strategy. And the third is that we have a website called "HablemosdePrEP.com" where we give complete information about what PrEP is, what the protocol is, and where they should go to make an appointment to start PrEP.

Key Informant Interview #8

<u>Personalized, Humanistic Care</u>

M: And in your ideal, how would you like for them to pass on information about prevention?
P6: Just like right now.
M: That they go and get you?
P6: Because otherwise I wouldn't be able to work because...
M: So that they don't bother you because otherwise the client will go. So, here is ideal because we can have confession…
P6: Yeah, it feels comfortable here.

FGD Participant #6 & Moderator

P5: I worked for a time in the Ministry of Health, in the area of Health Promotion. […] But yeah, I did not agree with many of the ways we were using to reach people with the information, right? And more if you are in the place they are working…
M: Exactly that point, how would you like it to be?
P5: Damn...more personal, right? I mean, yes, come and say: "Look, I'll leave this information here with you." Many times, though, we are so careless that we do not even read the information, but it should be done as we are doing now [in the focus group] because it is more private...you can have more confidence in saying something compared to on the street. Because, generally, I am a little more reserved, right? To me, I don't like

	that everyone will see me there...I don't know, they are very personal things…<br>FGD Participant #5 & Moderator<br><br><u>Organic Transmission (Word of Mouth)</u><br><br>*Well, for some it was through word of mouth. That is, a guy who is a sex worker came to the clinic to get tested as he would regularly, and he realized that downstairs, there is a sign that says "ImPrEP". He asks about it in counseling, they give him the information, they do the tests, they see that he is a candidate for PrEP, and they enter him into the protocol. Perhaps he, already having started PrEP, tells his friends that at Clínica Condesa they are giving PrEP. Perhaps this is how the spread of information was achieved.*<br>Key Informant Interview #1<br><br>*For others, I have told them, "Look, you are already taking it. You have more friends who do this type of work. Tell them to come." And yes, they have brought others to enter the program. Also, on social networks, they find that PrEP is becoming more common. So, they come to the clinic directly asking what it is because they saw it in a magazine, they saw it in an interview, they saw it on a website, they heard from a friend who is already taking PrEP and knows where to get it. So, they come with this idea that they need it.*<br>Key Informant Interview #3<br><br>*The vast majority of the answers...when we ask, "How did you find out about PrEP?" ...the vast majority tell you that they learned about it through someone they know who uses PrEP. Most have read something on social media about PrEP, but by reading it on social media, they do not get a very good idea of what PrEP is. When they ask their friends who already use it, then they become clear about the method and where they can get it.*<br>Key Informant Interview #2<br><br>*The first is the advantage that we have because we already had a Punto Seguro project that was directed towards male sex workers. So, this advantage was what facilitated things. Some of them [MSWs] already knew us. They were coming for their follow-up for other protocols or projects, and they got to know the clinic, the staff, and the service. So, that facilitated the opportunity where some of us could say: "Now there is the ImPrEP Mexico project. There is a PrEP Seguro project." They trust the information we have given them, and this has also facilitated things.*<br>Key Informant Interview #7<br><br>*Currently, I can tell you that they are the two most successful are by*

	word of mouth and by now coming to the clinic on a regular basis because few have come because of what they saw on Facebook or on social media. Generally, those who came were because they had already been tested for HIV here at the clinic.<br>Key Informant Interview #2<br><br>*Well, I think that one has been that, by coming to take the test directly and identifying them as candidates and explaining to them what it is about and why they are at constant risk…that had been a method that has been effective. Another that happens a lot is that many perceive themselves as subjects at risk and they come requesting this possibility.*<br>Key Informant Interview #3<br><br>*And the other way they learn about ImPrEP is when they come here for follow-up...that is, their routine HIV or syphilis exams. Well, they come and may see some small announcement that we have there in the hallway, or when the counselor is delivering the results of their exams, they may say, "Maybe you can add this PrEP option to what you are doing for prevention." In total, it has been different mechanism that have been part of the promotion.*<br>Key Informant Interview #7<br><br>*Sure, I have been inviting friends to take PrEP. Some have even gone and realized they were [HIV] positive. That is, they go in with the idea that no, but in reality, they were. So, they could not take PrEP, but that doesn't matter because it's more important that they take antiretrovirals. [...] it has even happened to me with two close friends. I invite them, and they tell me: "[...] I want to take PrEP." Well, look, I'll give you the number, his name is Edgar or Steven, contact them, they will give you an appointment [...]*<br>FGD Participant #2<br><br>*I have heard very little [about PrEP]. In reality, there is no information that I have seen on the internet or through advertising. It wasn't until I went to Clínica Condesa and saw there that it said "PrEP". What I have heard has been from acquaintances or from people who have told me that they take it, and the little I know from my ignorance is that it is to prevent the HIV virus.*<br>FGD Participant #1<br><br>*Well, I think everyone [should receive information about PrEP], right? I have friends who I tell, "Come on! Go [get tested]." And, why shouldn't they?*<br>FGD Participant #4<br><br>*M: And who would you tell that you are taking PrEP?*

	P5: To everyone. In fact, it's about trying to spread or get the message across in some way. And more than anything to the circle [of people] where I operate. [...] Right now, I'm with the gang on the street, and we are a bit reckless sometimes…<br>*M: Ok, so would you share it with everyone?*<br>*P5: With everyone, of course, and without any reservations like that.*<br>FGD Participant #5 & Moderator<br><br><u>In-person Interaction and Outreach</u><br><br>*Some of us who are part of the work team obviously know the gay community because we are part of the gay community. From the regularity of going to those spaces, we are familiar with the areas where sex work is regularly executed. So, what we have done is field work where, really, we identify the spaces, and we approach them. Some [of the MSWs] may be friends, and that makes it very easy for us to go to the space and locate ourselves [...]. So, that is the field. We go, and we invite them to the intervention.*<br>Key Informant Interview #7<br><br>*Something that would work would be approaching people in the places where they work...that is, all areas of sex work, whether they men, women, transgender [...]. Yes, face to face, personally because you feel more secure. It's like when Edgar approaches us on the street and says to you: "Hey, I want to invite you to a PrEP program, have you already heard about it?". And you start to open up, that is, I start to open up about personal topics, and I trust that I can talk to him, right? "Hey, I'm actually positive; hey, I'm actually negative. Yes, I am interested in it." That's what I mean, there is more trust approaching you in person.*<br>FGD Participant #2<br><br>*P4: There are people who go and tell you [about HIV prevention services] and even give you the address [of the facility]. I have met people from the Clínica Condesa who tell you: "Look, I will pay for your transportation, so you can get checked. When should I come for you?"*<br>*M: And you like that? Do you think it's okay?*<br>*P4: Yes.*<br>FGD Participant #4 & Moderator
RE-AIM Domain: Adoption	
Barriers to *Adoption*	<u>Lack of Public Policy</u><br><br>*First, it is not a public policy. Right now, ImPrEP is a protocol that allows only a number of participants to enter. We want to have more*

	evidence to propose that to the state that a public policy be made…to see if the state adopts it, modifies some laws or financing, and then offers it to the entire population. But I do see how that can be a bit of a complicated job. If we want to make public policies, we would have to show the benefits of having it and providing it. PrEP has been shown to be beneficial in other countries, but here other than that, we also have to consider if the health system is prepared to sustain it, especially because of its costs. Because first, you are going to give medication to people who have good health, who do not have HIV, who are HIV-negative. We must try to find political will and change the way they think. For civil organizations or health institutions, we will depend on that: that the state assumes the responsibility and that the state makes purchases and that the state provides tools such as PrEP.<br>Key Informant Interview #7<br><br><u>PrEP-, HIV-, and Sexual Minority-related Stigma</u><br><br>*For example, in many cases in Mexico, there are health personnel who do not know how to address the issue of sexuality in people. They don't know how to talk about sexual practices among men because there is stigma and there are prejudices. Still, I say that, in the public service, unless they are services like Clínica Condesa, which is a staff that has been trained for years…but this is not the reality of the entire health system. However, in private institutions, we do know of doctors who have this sensitivity.*<br>Key Informant Interview #6<br><br>*Yes, I think that would be the biggest difficulty we would face with the centers that would not distribute PrEP. I anticipate that would not be because they are not eligible, but rather, they would not accept it because of this issue of stigma…of prejudice. I think the health staff still needs more training, more awareness of the issues. Well, a little in general also the places where they are ... I think it would be highly complicated in areas where prejudice also permeates the entire culture of that area. There, people would not approach the center...that is a bit of difficulty that CAPACITS experience in other states of the country. Many people do not approach these places because they are so small, and the town is so big that everyone knows who is coming, who is going, who is taking the medicine, and I think that a little at the beginning that is what would happen with PrEP when you want to implement it in other states.*<br>Key Informant Interview #9<br><br>*We are still a country with a lot of stigma in health services. There are people who look at PrEP as the "Green Card" so that everyone has unprotected relationships. They have not understood the importance of PrEP as a prevention tool. They believe that giving PrEP is going to*

open sexual debauchery and open unprotectedness. And there are people who for moral or for cultural issues are detractors of PrEP.

Key Informant Interview #8

Absolutely, yes, and especially towards the community of trans women...a lot of prejudice, a lot of ignorance, a lot of stigma and discrimination. Not only in health services, but in jobs, universities, schools, in their own families. That is a challenge in Mexico. And the same happens for the gay community and men who have sex with other men. There is a lot of taboo about it. There are some circles in Mexico that have managed to eliminate that, but on the whole, Mexico is a country with a culture of much homophobia, transphobia, etc.

Key Informant Interview #4

Limitations of ImPrEP México

Because it is a real-life implementation protocol, it has things that have to be in common in Peru, in Brazil, and in Mexico. There are very few things that have to be common in all three countries. Because of this, there are many restrictions on access, and it is focused only on MSM and the population of trans women. We in Mexico tried to open it to serodiscordant couples: heterosexual men with a serodiscordant couple, women with a serodiscordant couple. But, we cannot put this into this protocol, but here in the clinic we have many situations where we diagnose a person that is part of a couple, and the other is at high-risk of being infected. But, we cannot put them into the protocol because it is a heterosexual person, either a man or a woman. So, they are not in ImPrEP. But, right now, we are only limited to that population: MSM and trans women. That is why we have these restrictions: because it is an implementation protocol in the 3 countries. When this phase ends, when the protocol ends, when this implementation intervention ends, then we think that the other cities also have to open it to other populations. But opening it means that it be adopted by IMSS, adopted by ISSSTE, and adopted by the other health systems.

Key Informant Interview #2

Clínica Condesa, I tell you, is very user-centered, and we try to do a different type of intervention... a differentiated attention for each person. In contrast, the ImPrEP protocol is the same for everyone and is very rigid. So, initially, it cost us a lot of work. Right now, we are more adapted. And, this is a process that must be made known to the user—that it is an intervention with characteristics of research that are very rigid times, that are scheduled appointments, that are more controlled interventions.

Key Informant Interview #2

Facilitators to *Adoption*	<u>NGOs</u><br><br>*For example, the context is that these organizations were already doing HIV testing for a long time; they were already giving assistance of prophylaxis for HIV. They were already giving out condoms. They were doing promotion—diffusion of prevention methods. They already had assistance for issues of human rights. They were already doing some trainings, principally in the population of MSM. And others were doing them with trans women and MSM. Therefore, with that experience, the only thing that was added was the service of distributing the medication of PrEP because the doctors of these organizations manage, the majority of the time, sexually-transmitted infections.*<br>Key Informant Interview #2<br><br>*It was thought to include organizations of the civil society that have already been recognized for their impact within the communities they are in. For example, in Mexico City, two civil organizations were chosen. One is called México Vivo. The other is called Inspira. They were the ones that have demonstrated that they serve a well-sized population, that they are within people's radar…in people's minds when they talk about friendly civil associations. Inspira and México Vivo are very attached to the community here in Mexico City. That is to say, at events, parties, and other things. In Guadalajara, there is one called CHECCOS that has been working in HIV for twenty or thirty-something years. In Puerto Vallarta, there is one called SETAC that has also done a lot of field work. Therefore, these civil organizations were chosen as a way to bring PrEP closer to civil organizations that also work directly and constantly with the population.*<br>Key Informant Interview #3<br><br><u>Sensitized Providers</u><br><br>*The first providers we invited to ImPrEP had already worked with the clinic [Clínica Condesa] and demonstrated that they have worked with the community. They have experience doing tests, counseling. They are people who have not had problems with the LGBT [community], with sexuality. From the psychologist, the nurse, the doctor, and the social worker, it is evident that they do not have problems [with this work] and that they treat the patient well as a human being. They care for the patient as the patient wants to be cared for in that moment. They have empathy towards that other person.*<br>Key Informant Interview #7<br><br>*In Mexico, we think that, to expand the program at all levels, the best profile is a general practitioner. That is the best profile for the costs*

	associated with contracts, also for the type of users that you are going to be seeing, and we definitely need personnel and providers that are sensitized with the populations… that are sensitized with MSWs, with MSM, with transgender women. Because of the experience we have had with treating HIV, we know that if providers are not sensitive to these populations, there are many barriers between the doctor and patient. And, that is not good.<br>Key Informant Interview #2<br><br><u>Existing Infrastructure at the National Level and Clinic Level</u><br><br>*I think it is important to not rule out organizations that do not have a screening center. There are civil organizations that for a long time do not offer rapid tests but are the link between a population that is in a very distant area or in a very complicated context. What they do is be a link or support that the person who is far from being able to reach the detection center so that they reach the detection center. There are civil organizations that do this direct work with the populations that could do this issue of education and generate the link between people who are at risk of acquiring HIV and the organization that can give PrEP.*<br>Key Informant Interview #6<br><br>*What worked with the workers for recruitment was that it was fast. That it wouldn't take you long. Suddenly there were processes during the project that became extensive. They began to make the processes more extensive so that the person received their first bottle. So, the less you make people wait, it is better…a better disposition of them in every way. When the PrEP service was in Inspira, people did not wait. When the service was moved to Clínica Condesa, there were complaints on social media. They said that for a consultation that normally takes an hour or two hours maximum, they had to wait more than 5 hours in the clinic to get their medication. So, the faster the process, people will have more disposition and more retention.*<br>Key Informant Interview #5
CROSS-CUTTING THEMES FOR *REACH* AND *ADOPTION*	
Demand for PrEP	<u>High Patient Demand</u><br><br>*During the implementation, let's say that the operational coordination established a goal with each civil organization according to the installed capacity. They assigned each civil organization a number of users to receive PrEP. So, in Inspira, 101 people entered PrEP in that period of time. However, Inspira had a waiting list at that time of more than 300*

	people, but only 100 could enter. In each civil organization, a contract was made, and goals were set for what had to be met. With each organization the goal of entering people into PrEP was different. In Inspira, it was 101 that received PrEP, but the waiting list was more than 300.<br>Key Informant Interview #6<br><br>*Surely, there are already organizations of the civil society [NGOs] that are offering PrEP outside of the ImPrEP protocol because of that same experience. If they were able to give antiretroviral treatment to people living with HIV when they could not get it in health services in the past, then I have no doubt that they are already doing it with PrEP in some way.*<br>Key Informant Interview #7<br><br>*M: So, I'll ask you again, are you willing to do the screening test [for HIV] to get PrEP, considering that it is free?*<br>*P5: Totally…yes, I do want it, please.*<br>*P6: Yes, without a doubt…whatever happens will happen.*<br>*P7: Same here, yes.*<br>*P4: Me too…*<br>*P8: Different. I'd rather not.*<br>FGD Participant #4, #5, #6, #7, #8 & Moderator<br><br>*I think being informed [about HIV and other STI] is important…and prevention, prevention at all levels, no? Children, adolescents, adults. I think there is still a lot of stigma regarding this, and it is very important that all sectors address it…at the school level, family level…So, for me, yes, this question of information is very important. It is important to share it, not only about HIV, but all illnesses that exist.*<br>FGD Participant #1<br><br>*M: Who should receive information about PrEP? And how should that information be given?*<br>*P5: I say that everyone should…starting in elementary schools. That is, if they really want there to be an orientation toward young people, more than anything, it should be before they start having sex…because today, the youth is not the same as when I was growing up. […] The age is increasingly younger when they start having sex…girls, boys, everyone, nowadays it is even.*<br>*M: And how should this information be presented?*<br>*P5: Well, through social networks, health fairs…for example, in the health fairs where they show the correct use of condoms, by the Government of Mexico City. […] I do not understand why if this [PrEP] is known, if there is knowledge that it can really prevent HIV…it seems*

	absurd to me that not even the government is doing anything to stop something that has done much damage throughout many years.<br>FGD Participant #5 & Moderator<br><br>*Well everyone, from children who have it well in their head what HIV is and what can happen if you are infected or how they can prevent it and [spreading this information] through schools, social media, television, brochures, radio…whichever.*<br>FGD Participant #6
